# Laser-Induced Photothermal Pulling of Dyed Droplets on a Superhydrophobic Surface

**DOI:** 10.1021/acs.langmuir.5c00160

**Published:** 2025-04-29

**Authors:** Peiying Han, Zhaofei Zhu, Zoran M. Cenev, Ville Liimatainen, Heng Zhang, Bo Chang, Quan Zhou

**Affiliations:** † School of Mechanical and Electrical Engineering, Shaanxi University of Science and Technology, Xi’an, Shaanxi 710021, China; ‡ Department of Electrical Engineering and Automation, School of Electrical Engineering, Aalto University, Espoo 02150, Finland

## Abstract

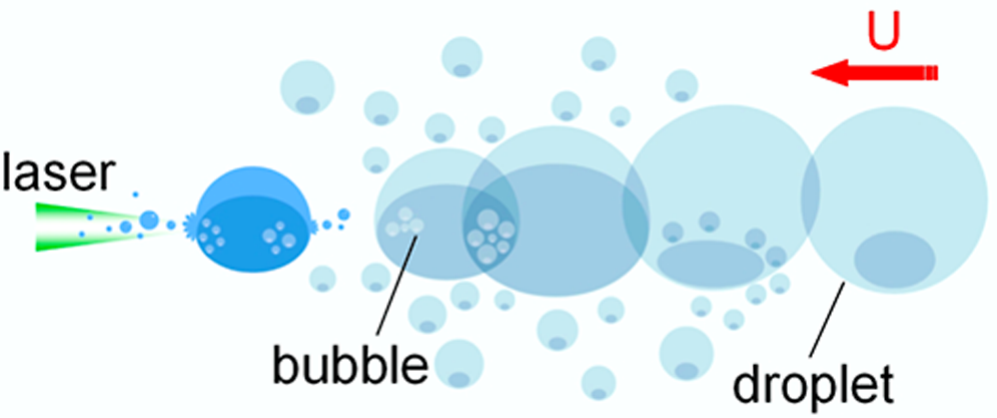

Understanding the interaction between laser beams and liquid droplets has significant implications for applications in microfluidics and optical manipulation. Laser beams have previously been reported to act as tractor beams to pull microscopic particles toward the light source or serve as a heating source when directed from above to induce lateral droplet motion via photothermal effects. However, it remains unknown whether a laser beam can move a droplet toward its source. In this study, we show that a laser beam can pull a dyed droplet on a superhydrophobic surface toward the light source through a sequence of photothermal effects. By directing a green laser beam near the bottom front of a dyed droplet, we observe that the droplet moves toward the light source in two distinct stages. Initially, the dyed droplet advances due to contact angle hysteresis and coalescence with condensation satellite droplets. Subsequently, the droplet motion is stimulated by iterative bubble bursting, coalescence, and relaxation, a combination of effects not reported earlier. We experimentally investigate this motion phenomenon and analyze the influence of laser power and focal point position on droplet motion, offering new insights into laser-induced droplet manipulation.

## Introduction

Light beams have long been imagined to be able to pull objects toward the sources. Such phenomena, conceptually introduced in the movie Star Wars and termed tractor beams, have been observed later at microscopic scales. Particle motion under the influence of a tractor beam may be induced by optical scattering,^1,2^ gradient forces,^3,4^ or a combination of both,^5,6^ as well as photophoretic forces.^7,8^ However, all the reported experiments are for solid micro- and nanoparticles, from hundreds of nanometers up to tens of micrometers.^1−8^

Although laser beams have not previously been shown to pull macroscopic objects, when directed from above, they can effectively move water or metal droplets via photothermal effects. Various mechanisms for moving the triple-phase contact line have been proposed, including coalescence with condensation satellite droplets,^9,10^ and local change of surface energy.^11,12^ Focused infrared laser irradiation was shown to induce a state transition of a water droplet on a superhydrophobic surface from Cassie to Wenzel, facilitated by the expansion of the triple-phase contact line through the coalescence with condensed satellite droplets under the photothermal effect.^9^ A laser can also heat the front edge of water containing gold nanoparticles in a microfluidic channel and expand the triple-phase contact line through evaporation and coalescence with satellite droplets.^10^ Additionally, a laser beam can reduce the surface tension of the oil–air interface of a substrate, leading to droplet motion,^11^ and induce wettability changes on the substrate to trap metal droplets along the optical axis.^12^ Despite all the impressive work, it remains a question if a laser beam can move a droplet toward the laser source.

In this article, we present a motion phenomenon involving laser-induced photothermal pulling of dyed droplets on a superhydrophobic surface, providing new insights into controlled droplet manipulation mechanisms. Utilizing a 520 nm laser with a power output of 458 mW focused on the leading edge of a dyed droplet, we initiated a sequence of photothermal effects. These effects encompass a progressive series of phenomena, including changes in the contact angle, consolidation, and coalescence of satellite droplets, and the formation and subsequent bursting of air bubbles, all of which contribute to the propulsion of the dyed droplet toward the laser source, covering a distance nearly twice its initial size.

## Results

### Behavior of Dyed Droplets under Focused Laser Irradiation

**Figure 1:**
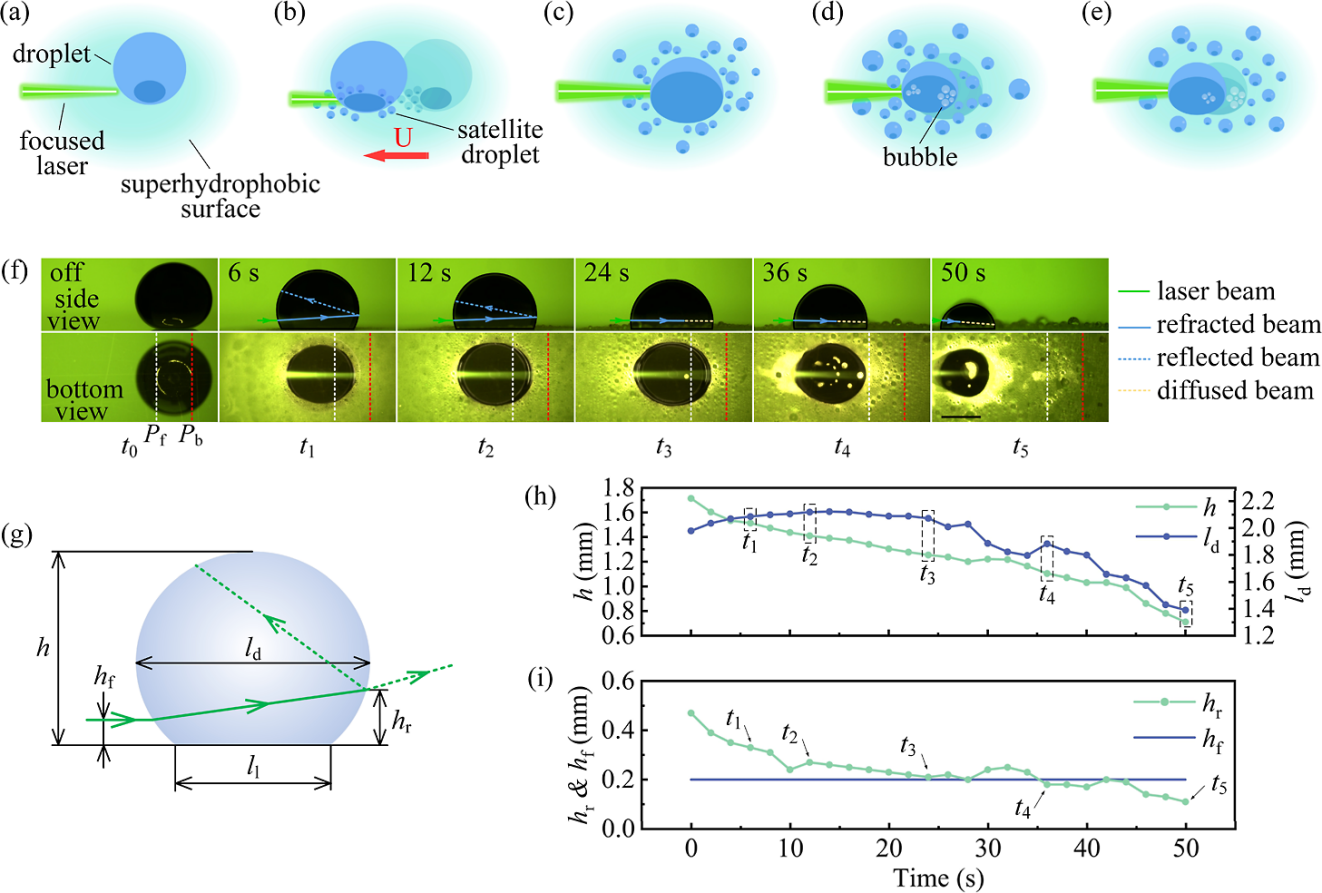
Laser-induced photothermal pulling of a dyed droplet (illustration not to scale). (a) Focused laser beam is directed near the bottom position of the droplet on a superhydrophobic surface. (b) Satellite droplets condense around the main droplet, largely near the advancing contact line of the main droplet, causing the main droplet to coalesce with the satellite droplets and move forward. The red arrow indicates the direction of droplet motion. (c) Condensation droplets increasingly form around the expanding triple-phase contact line of the main droplet. (d) Upon further laser irradiation, bubbles form near the solid–liquid interface of the main droplet, while the condensation droplets keep enlarging. (e) Bubbles iteratively form and burst, propelling the droplet toward the light source. (f) Evolution of the laser path inside the droplet. *p*_f_ and *p*_b_ represent the initial positions of the front and back of the droplet–substrate contact line, respectively. The scale bar is 1 mm. (g) Parameters of the laser path within a droplet. *l*_l_ is the long axis of the solid–liquid interface of the droplet. *h*_r_ is the distance between the hitting point of the refracted light on the back of the droplet and the substrate within the droplet. *h*_f_ is the distance between the hitting point of the laser beam on the front of the droplet and the substrate. (h) Evolution of the height *h* and long axis *l*_d_ of the droplet. *t*_1_–*t*_5_ correspond to different moments in subfigure (f). (i) Evolution of *h*_r_.

The motion phenomenon is illustrated in Figure 1. By irradiating the advancing side of the dyed droplet placed on a superhydrophobic surface using a focused laser beam (Figure 1a), the advancing contact angle θ_a_ of the droplet reduces, followed by the droplet evaporation and condensation forming satellite droplets on the substrate, pulling the droplet toward the light source (Figure 1b). Further laser irradiation leads to additional coalescences with the surrounding satellite droplets, expanding the triple-phase contact line and resulting in reduced net motion (Figure 1c). However, prolonged laser irradiation raises the temperature of the dyed droplet to near boiling point, forming bubbles inside the droplet near the liquid–solid interface (Figure 1d). These bubbles iteratively form and burst, causing the dyed droplet to coalesce with the satellite droplets followed by relaxation, pulling the droplet toward the light source (Figure 1e).

When a focused laser beam irradiates the dyed droplet surface, refraction and reflection of the laser beam occur within the droplet. The spatiotemporal evolution of the path of the laser beam inside the dyed droplet plays an important role in creating a series of photothermal effects that drive the droplet toward the light source. To demonstrate the phenomenon, we dispensed a 3.5 ± 0.2 μL dyed water droplet onto a superhydrophobic surface, directed a 458 mW laser beam (wavelength 520 nm) slightly above the triple-phase contact line, and observed the droplet moving toward the beam. The light path in this study was derived based on theoretical estimations using the refractive indices of air and water, as direct observation of the light path is not possible from the side-view camera. As shown in Figure 1f, when a laser beam hit the front surface of a dyed droplet at a height (*h*_f_), the beam was refracted and propagated inside the droplet. A portion of this refracted beam was then internally reflected at the back of the droplet or refracted out of the droplet (Figure 1g). Upon continuous laser irradiation for 12 s, the solid–liquid interface of the droplet expanded progressively (Figure 1f, *t*_2_). This, in turn, decreased the droplet height *h* from the initial 1.7 to 1.4 mm and increased the long axis of the droplet *l*_d_ from the initial 2 to 2.1 mm (Figure 1h, *t*_0_–*t*_2_). Correspondingly, *h*_r_, the distance between the hitting point of the refracted light on the back of the droplet and the substrate, decreased from the initial 0.5 to 0.3 mm (Figure 1i, *t*_2_). After 24 s at *t*_3_, *h*_r_ decreased to about 0.2 mm (Figure 1i, *t*_3_), resulting in more beam irradiating near the solid–liquid interface of the droplet (Figure 1f, *t*_3_). With further laser irradiation, *h*_r_ kept around 0.2 mm (Figure 1i, *t*_3_–*t*_5_). However, bubbles appeared near the solid–liquid interface of the droplet after 24 s at *t*_3_ (Figure 1f, *t*_3_). Consequently, the refracted laser beam showed significant scattering by the air bubbles near the solid–liquid interface, weakening the beam, which initially bent upward and then downward due to the change of the front curvature of the droplet with decreasing volume (Figure 1f–i, *t*_3_–*t*_5_). The example shown in Figure 1 is typical; more light path evolution data showing similar processes can be found in Figures S1 and S2.

### Motion Induced by Contact Angle Hysteresis and Coalescence

**Figure 2:**
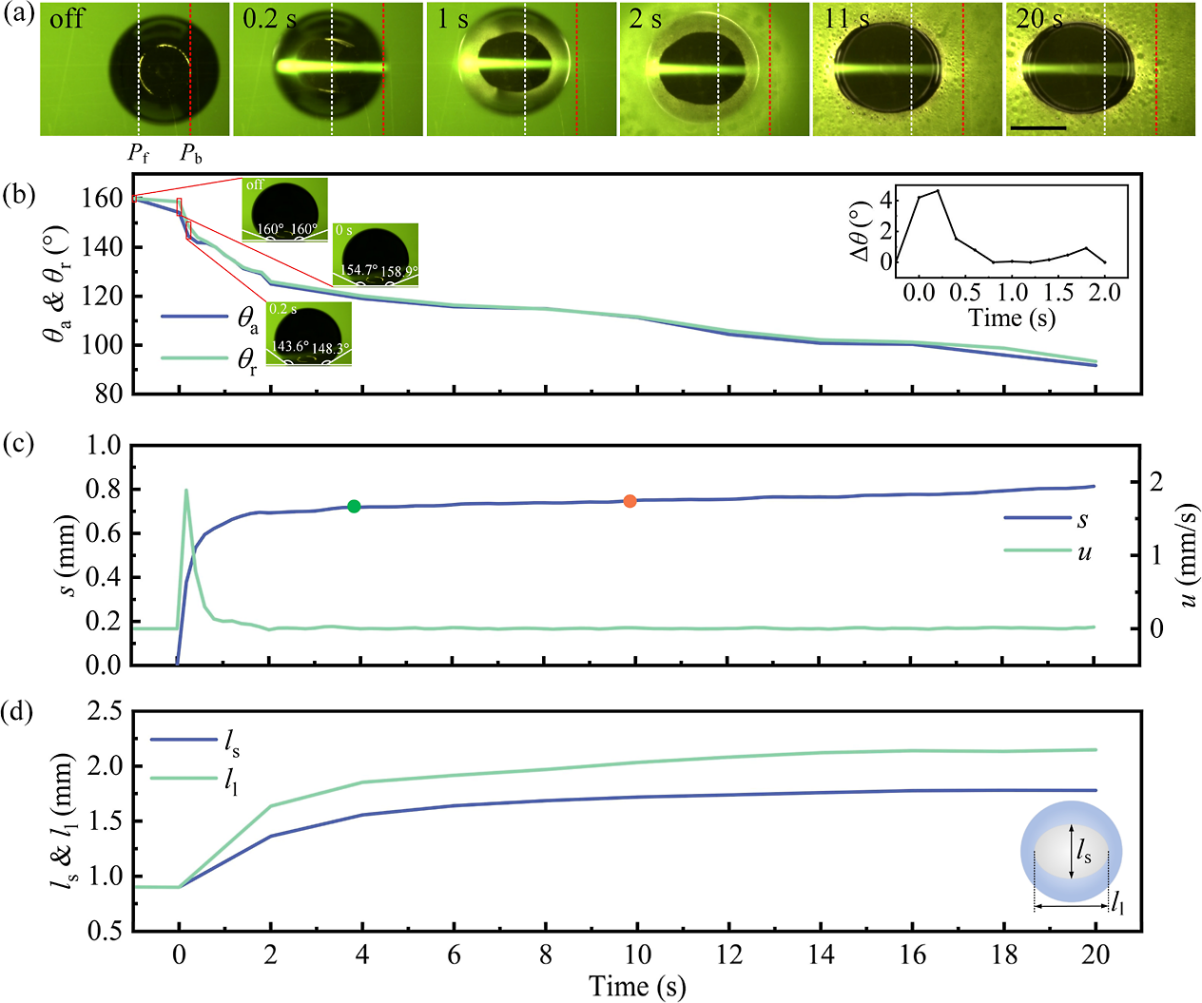
Droplet motion induced by contact angle hysteresis and coalescence. (a) Bottom-view images of the experiment, showing the expansion of the triple-phase contact line of the main droplet and the growth of the condensed droplets. *p*_f_ and *p*_b_ represent the initial positions of the front and back of the droplet-substrate contact line, respectively. The scale bar is 1 mm. (b) Temporal evolution of the contact angle of the droplet. Here, θ_a_ and θ_r_ are the advancing and receding contact angles of the droplet, respectively. Δθ represents the contact angle hysteresis of the droplet, Δθ = θ_r_ – θ_a_. (c) Displacement *s* and velocity *u* of the droplet through coalescence. Here, the green dot and orange dot represent the response time and the settling time, respectively. (d) Temporal evolution of the shape of the triple-phase contact line of the droplet. Here, *l*_s_ and *l*_l_ are the short and long axes of the triple-phase contact line of the droplet, respectively.

During the initial 20 s of laser irradiation, the dyed droplet motion was primarily caused by thermally induced contact angle hysteresis, as well as coalescence between the satellite droplets and the main droplet from thermally induced evaporation and condensation. Detailed analysis of this phase is illustrated in Figure 2.

Upon laser irradiation at 0 s, the entire droplet moved toward the light source (Figure 2a). The advancing contact angle θ_a_ and the receding contact angle θ_r_ decreased from the equilibrium contact angle θ_e_ of 160° to 154.7° and 158.9°, respectively (Figure 2b, see the inset of Figure 2b). At 0.2 s, the contact angle hysteresis Δθ increased to 4.7°, and the displacement of the droplet *s* increased to 0.5 mm (Figure 2c). Since *h*_f_ is rather lower, this suggests that the droplet absorbed much energy at the spot near the advancing contact line, leading to a smaller θ_a_ compared to the θ_r_ (Figure 2b). However, Δθ quickly reduced to 0° within about 0.8 s (see the inset of Figure 2b).

After the first motion, satellite droplets formed around the main droplet, more in the front (Figure 2a, see Video S1), reducing the droplet velocity *u*. The coalescence of the main droplet with the satellite droplets led to an expansion of the triple-phase contact line of the main droplet (Figure 2d). Since there were more condensed droplets in the front, the main droplet showed a net motion toward the laser source with the velocity *u* less than 0.2 mm/s after 2 s, as shown in Figure 2c. We attribute the initial front-biased evaporation and condensation to a lower *h*_f_ and a high *h*_r_ (Figure 1i), causing the photothermal energy to be absorbed more in the low front of the dyed droplet. Additionally, as shown in Figure 2c, the droplet reached a response time of 3.8 s and a settling time of 9.8 s during its motion.

When the droplet was continuously irradiated by the laser for 2 s, both the short axis *l*_s_ and the long axis *l*_l_ of the triple-phase contact line of the droplet expanded from the initial 0.9 to 1.4 and 1.6 mm, respectively (Figure 2d), and the ratio of the *l*_s_ to *l*_l_ is 0.88. Meanwhile, both the θ_a_ and the θ_r_ of the droplet decreased to 127.2° (Figure 2b), resulting in a reduction in droplet velocity *u* (Figure 2c). We attribute the diminishing of Δθ to the thermal equilibrium caused by thermal convection inside the droplet. Additionally, we observed a gradual blurring of the solid–liquid interface of the droplet (Figure 2a, see Video S1). This phenomenon can be attributed to the main droplet undergoing a Cassie-to-Wenzel transition, similar to the previous observation of the expansion of the triple-phase contact line under top laser irradiation.^13,14^

With continuous laser irradiation, the condensation droplets grew over time. As shown in Figure 2a, when the laser irradiated the main droplet for 20 s, the maximum size of the condensation droplets was about 0.1 mm around the triple-phase contact line of the main droplet (Figure S3). This reduced the pulling of the condensation droplets on the main droplet and the velocity *u* of the main droplet. Such a phenomenon is similar to the coalescence of unequal droplets, where the larger droplet is drawn toward the smaller one, with the pull increasing as the size of the smaller droplet decreases.^15−17^ Additional experimental data on this phase can be found in Figure S4, showing similar processes.

### Motion Caused by Bubbles

**Figure 3:**
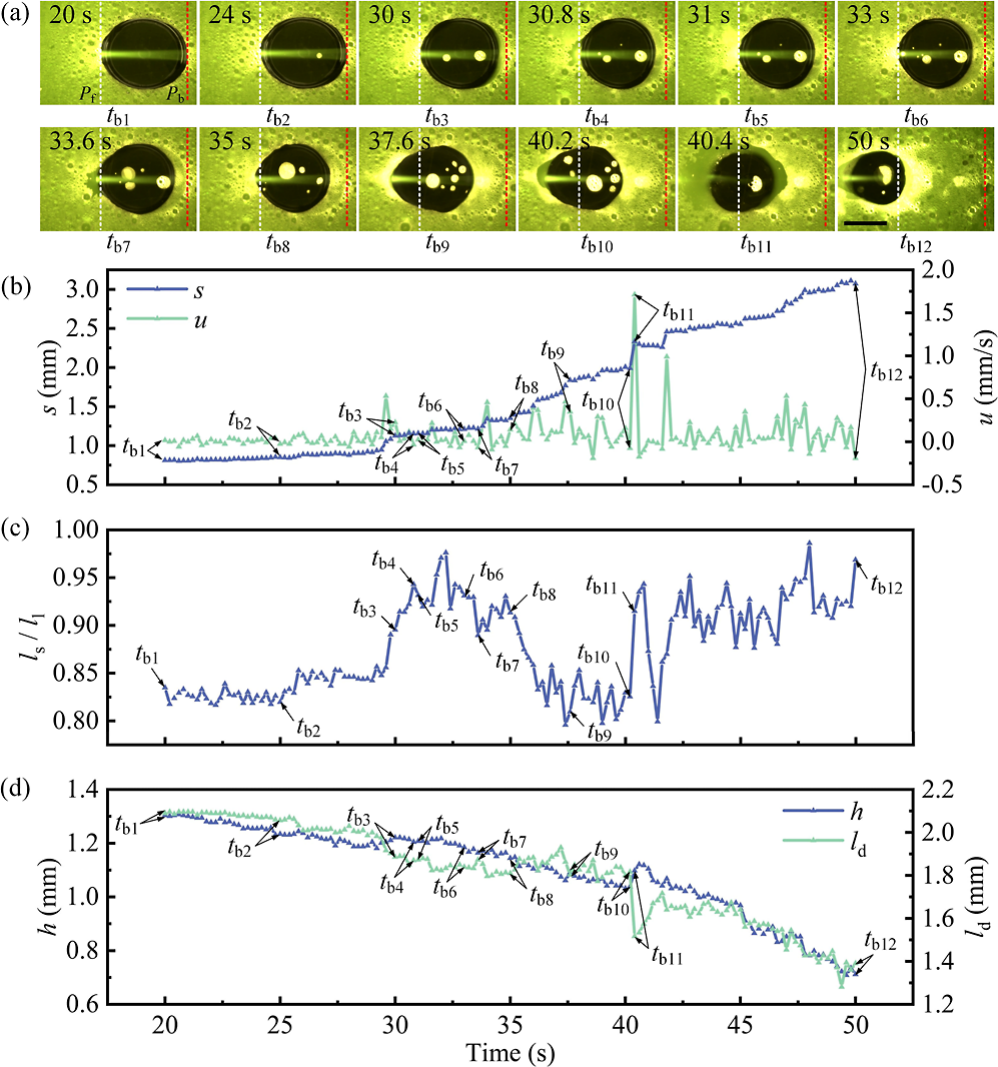
Droplet motion caused by bubble bursting and relaxation. (a) Bottom-view images of the experiment at different moments, with *t*_b1_–*t*_b6_ and *t*_b7_–*t*_b12_ denoting the first and second stages, respectively. *p*_f_ and *p*_b_ represent the initial positions of the front and back of the droplet-substrate contact line, respectively. The scale bar is 1 mm. (b) Displacement *s* and velocity *u* of the droplet during motion. Time points *t*_b1_–*t*_b12_ correspond to different moments in subfigure (a). (c) Temporal evolution in the ratio of the short axis *l*_s_ to the long axis *l*_l_ of the triple-phase contact line of the droplet. (d) Temporal evolution in the height *h* and long axis *l*_d_ of the droplet.

After 20 s of laser radiation, during *t*_3_–*t*_5_ in Figure 1, the motion of the dyed droplet was mainly caused by the relaxation of the droplet and the asymmetric droplet dynamics resulting from laser-induced boiling. The pulling process can be divided into two stages. The first stage involves only the receding contact line of the droplet being pulled. The second stage sees the whole droplet being pulled toward the light source. The details of this part are further analyzed and are illustrated in Figure 3.

In the first stage (20–33 s), the receding contact line of the droplet moved significantly toward the light source compared to the advancing contact line. As shown in *t*_b1_–*t*_b2_ of Figure 3a, the droplet remained stationary for a few seconds after being irradiated by the laser for 20 s. At 24 s (*t*_b2_), visible bubbles appeared at the rear side of the solid–liquid interface of the droplet. We attribute this to *h*_r_ being lower than *h*_f_ (Figure 1i, *t*_3_), leading to greater heating near the rear side of the solid–liquid interface. After 30 s (*t*_b3_), the receding contact line of the droplet was pulled toward the light source, while the advancing contact line hardly moved. Simultaneously, the velocity *u* of the droplet increased from 0 at 20 s to 0.2 mm/s (Figure 3b, *t*_b3_), and *l*_s_/*l*_l_ increased from 0.83 to 0.9 (Figure 3c, *t*_b3_). The droplet continued to move toward the light source (Figure 3a, *t*_b4_–*t*_b6_) and *l*_s_/*l*_l_ = 0.93 at 33 s (Figure 3c, *t*_b6_). We attribute the tendency of the ratio *l*_s_/*l*_l_ approaching 0.98 to relaxation of the droplet. At 30.8 s, the bubbles burst at the front side of the droplet. Soon after, the bubbles burst from both the front and the back sides (Figure 3a, *t*_b4_–*t*_b6_, see Video S2). However, the bubbles burst more frequently at the front side compared with the rear, propelling the droplet forward (Table S1). During the bursting process, expanding air bubbles stretch the droplet and push the contact line outward until the water meniscus ruptures. Following the burst, the droplet enters a relaxation phase, during which surface tension acts to restore the deformed meniscus toward a spherical shape. This restoration process generates a net force that shifts the entire droplet in the direction of the bursting. When bursts occur on both the front and back sides of the droplet, the droplet typically moves toward the side experiencing a greater number of bursts.

In the second stage (33–50 s), we observed that the droplet was pulled toward the light source with both advancing and receding contact lines moving. At 33.6 s, the advancing contact line of the droplet moved forward (Figure 3a, *t*_b7_). Subsequently, the receding contact line of the droplet was pulled toward the light source (Figure 3a, *t*_b8_–*t*_b10_). The displacement of droplet *s* reached 2 mm at 40.2 s (Figure 3b, *t*_b10_). At 40.4 s, most bubbles within the main droplet burst from its front side, causing the advancing contact line to retract after coalescing with the condensed droplets (Figure 3a, *t*_b11_, Figure S5). At 50 s, we observed that both the advancing and the receding contact line of the droplet moved forward, and the displacement *s* reached 3.1 mm from 0.8 mm at 20 s (*t*_b1_) (Figure 3b, *t*_b1_–*t*_b12_).We attribute the forward motion of the whole droplet in a bursting–relaxation cycle to the receding contact line of the droplet being pulled toward the light source during the relaxation. As the laser continued irradiating the droplet, the bursting–relaxation process occurred iteratively until the droplet completely evaporated (Video S1).

We attribute the different motion behavior of the dyed droplet in the two stages to bubble influences on the laser light path. In the first stage (Figure 3a, *t*_b1_–*t*_b6_), the laser heated the most of the liquid–solid interface, and bubbles burst from both the front and back side of the droplet, while the number of bubbles bursting from the droplet was few. Since the volume of the droplet was relatively large (Figure 3d), this resulted in the force generated by the bubbles bursting within the droplet being insufficient to pull the entire droplet toward the light source (see Video S1). In the second stage (Figure 3a, *t*_b7_–*t*_b12_), a larger number of bubbles were generated in the droplet, hindering the transmission of the laser beam toward the rear side of the droplet, causing the front side of the droplet to absorb more heat. Therefore, the bubbles burst mostly from the front side of the droplet, resulting in the droplet being pulled toward the light source as a whole (see Video S2). Additional data can be found in Figure S6a, which illustrates the distribution of the final displacement of the dyed droplets. Note that variations in total droplet displacement are influenced by surface inhomogeneities, such as defects and dust particles, which cause local pinning or deviations in the condensation process and motion path.

### Influences of Laser Parameters

#### Influence of Laser Power on Coalescence

**Figure 4:**
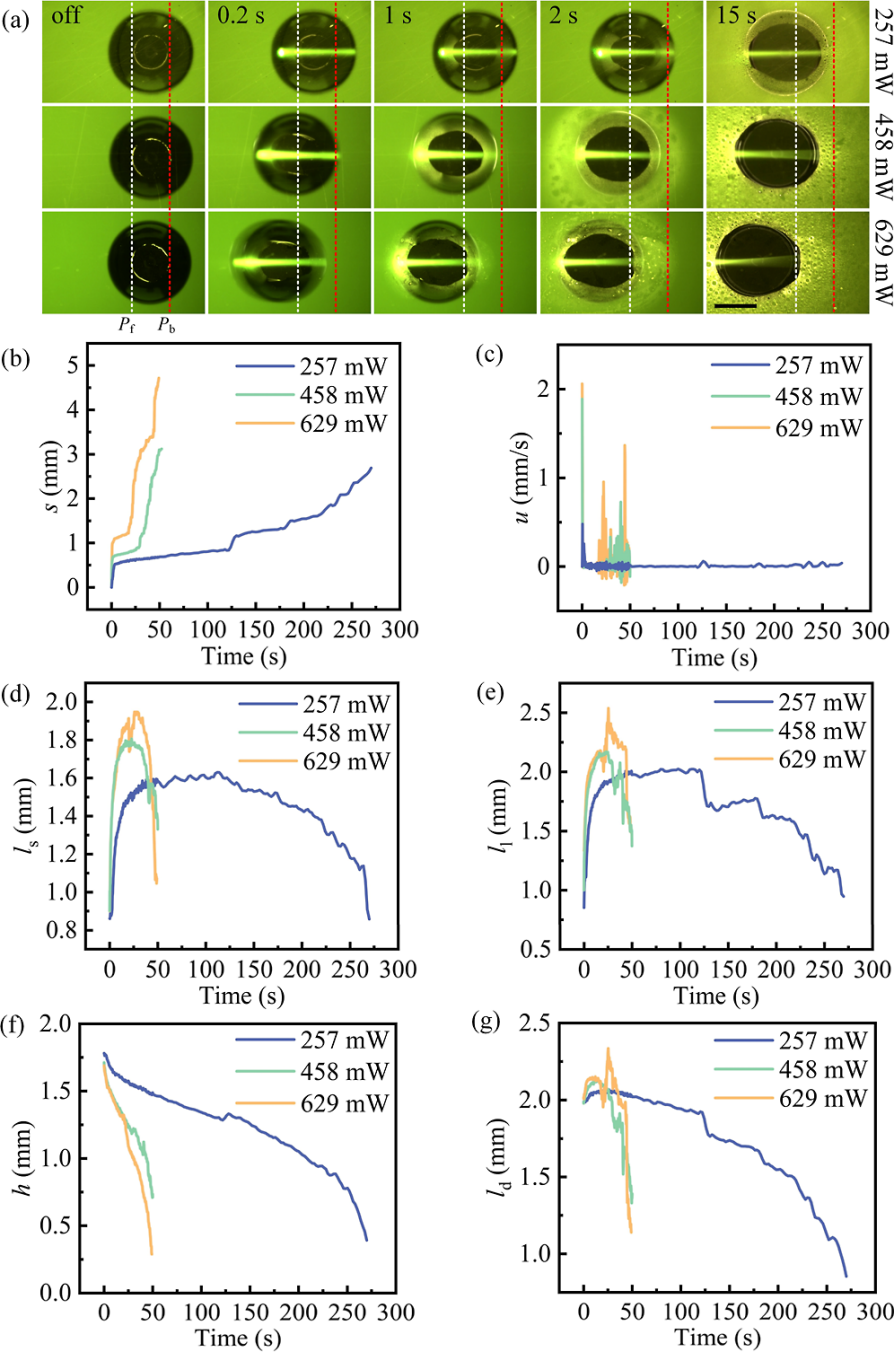
Influence of laser power on the dyed droplet. (a) Bottom-view images of the temporal evolution of the triple-phase contact line of the droplet from the experiment at different laser powers. *p*_f_ and *p*_b_ represent the initial positions of the front and back of the droplet-substrate contact line, respectively. The scale bar is 1 mm. (b) Temporal evolution of droplet displacement *s*. (c) Temporal evolution of droplet velocity *u*. (d) Temporal evolution of the short axis of the triple-phase contact line of the droplet *l*_s_. (e) Temporal evolution of the long axis of the triple-phase contact line of the droplet *l*_l_. (f) Temporal evolution of the height of the droplet *h*. (g) Temporal evolution of the long axis of the droplet *l*_d_.

The laser power used in the study was primarily 458 mW. However, the influence of laser power has been studied using three power settings: 257, 458, and 629 mW. We observed a positive correlation between laser power and both droplet velocity and displacement of continuous laser irradiation on the dyed droplet (Figure 4a–c). When the droplet was irradiated at different laser powers (257, 458, and 629 mW) for 0.2 s, only the droplet irradiated with the 257 mW laser remained almost stationary (Figure 4a). The displacements of the droplets *s* were 0, 0.4, and 0.4 mm, respectively (Figure 4b). Correspondingly, the velocities *u* were 0, 1.9, and 2.1 mm/s, respectively (Figure 4c). At 2 s, the displacements of the droplet *s* were 0.4, 0.7, and 1.1 mm, respectively (Figure 4b). Subsequently, the main droplet was slowly pulled toward the light source when it coalesced with condensate droplets (see Video S3). At 15 s, the displacements of the droplet *s* were 0.6, 0.8, and 1.2 mm, respectively (Figure 4b). When the droplet was continuously irradiated with a 257 mW laser for 256 s, it was drawn toward the light source under surface tension-induced relaxation, resulting in a displacement *s* of 2.4 mm (Figure 4b and Video S3).

An interesting phenomenon is that after the dyed droplet was irradiated by lasers of different powers (257, 458, and 629 mW) for 2 s, the receding contact line of the droplet remained almost stationary for 13 s, while the whole triple-phase contact line gradually expanded over time, as shown in Figure 4a. We attribute this to the droplet reaching thermal equilibrium under laser irradiation. Additionally, the greater the laser power, the faster the expansion of the triple-phase contact line of the droplet. Specifically, when the droplet was continuously irradiated by lasers of different powers for 15 s, the short axis *l*_s_ of the triple-phase contact line of the droplet increased from the initial 0.9 to 1.4, 1.8, and 1.9 mm, respectively (Figure 4d), and the long axis *l*_l_ increased from the initial 0.9 to 1.8, 2.1, and 2.2 mm, respectively (Figure 4e).

#### Influence of Laser Power on Bubble Formation and Bursting

After continuous irradiation, the dyed droplet can be pulled toward the light source due to bubbles bursting. For a laser power of 257 mW, this motion started at 256 s; for 458 mW, it started at 33 s; and for 629 mW, it started at 17.8 s. As shown in Figure 4b, the maximum displacements of the droplet (*s*) at different laser powers were 2.7, 3.1, and 4.7 mm, respectively. In addition, higher laser power led to earlier bubble formation at the solid–liquid interface of the droplet (Figure S7). Increased laser power also accelerated droplet volume reduction (Figure 4f–g), causing the refracted light beams within the droplet to move closer to the substrate (Figures 4a and S7). Notably, after 256 s of continuous irradiation with a 257 mW laser, bubbles formed at the solid–liquid interface of the droplet. However, the bursts did not significantly affect the droplet motion (see Video S3). Additional data and the distribution of the final displacement of dyed droplets can be found in Figure S6a–c and the means and deviations are shown in Figures S8 and S9. The experiment for the 458 mW case is also demonstrated in Video S1 and for 257 and 629 mW in Video S3. The jetting phenomena observed in these videos result from the coalescence of condensate droplets around the main droplet.^18−20^

### Laser Focusing Point

**Figure 5:**
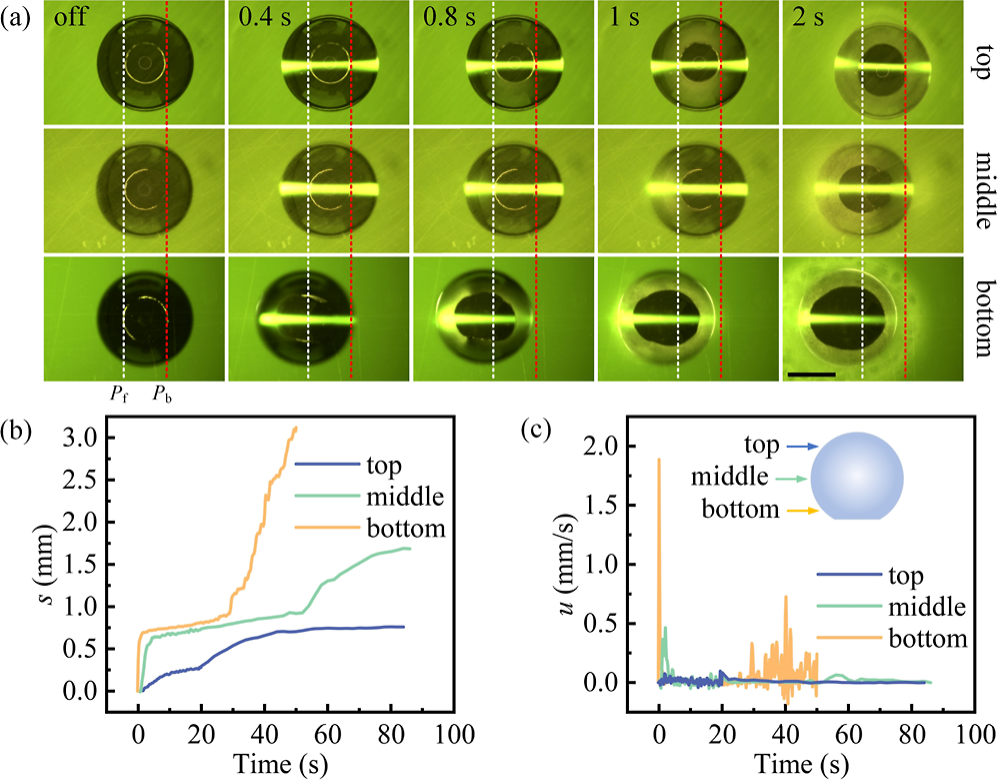
Influence of laser focusing point on the droplet. (a) Bottom-view images from the experiment under varying laser focusing point on the droplet. *p*_f_ and *p*_b_ represent the initial positions of the front and back of the droplet-substrate contact line, respectively. The scale bar is 1 mm. (b) Temporal evolution of the displacement of the droplet *s*. (c) Temporal evolution of the velocity of the droplet *u*.

We studied dyed droplet motion behavior with a 458 mW laser irradiating at different hitting point heights *h*_f_, near the top (*h*_f_ = 8/9*h*), middle (*h*_f_ = 1/2*h*), and bottom (*h*_f_ = 1/10*h*) of the droplet. Upon laser irradiation for 2 s, the greatest displacement was observed when *h*_f_ = 1/10*h* (Figure 5a). With continued laser irradiation, the final displacements *s* of the droplet were 0.8, 1.7, and 3.1 mm, respectively (Figure 5b). As shown in Figure 5c, the closer the focus was to the substrate, the greater the maximum velocity of the droplet. Specifically, the maximum velocity *u* of the droplet was greatest (1.9 mm/s) for *h*_f_ = 1/10*h*. We attribute the increase in droplet velocity *u* and travel distance *s* as the focal point approaches the substrate to the earlier formation of condensed satellite droplets near the front side of the main droplet. This earlier condensation reduces in the advancing contact angle θ_a_ sooner (see Video S4 and Figure S10), causing the droplet with a focal point height of *h*_f_ = 1/10*h* to move toward the light source first.

Interestingly, the droplet deviated from the optical axis for *h*_f_ = 8/9*h*. As shown in Figure 5a, when the droplet was irradiated at *h*_f_ = 8/9*h* for 1 s, the region around the triple-phase contact line of the main droplet was blurred by satellite droplets. At 2 s, the triple-phase contact line of the droplet expanded, and the droplet deviated from the optical axis (Figure S11). This phenomenon is attributed to the thermocapillary flow within the main droplet, leading to the nonuniform condensation of satellite droplets around the triple-phase contact line of the main droplet. Subsequently, the random coalescence of the main droplet with the satellite droplets results in a deviation of the main droplet from the optical axis. The experiment in Figure 5 is illustrative. The total droplet displacements at different focal positions (top, middle, bottom) are reported in Figure S6d,e,a, with their mean and standard deviations in Figure S12. The experiment for laser irradiation near the bottom of the droplet can be found in Video S1, and that for near the top and middle cases can be found in Video S4.

## Conclusions

In this study, we discovered that a focused laser beam can drive dyed droplets on superhydrophobic surfaces to move toward the laser source, following a dual-stage movement mechanism. Experimental results showed that satellite droplets first condense on the substrate at the front side of the main droplet irradiated by the light beam. Once the main droplet coalesced with the satellite droplets at its front side, it was pulled toward the light source. Subsequently, expansion of the triple-phase contact line increased resistance during droplet motion, reducing its velocity. Additionally, at laser powers of 458 and 629 mW, bubbles burst from the droplet surface, further pulling the droplet toward the light source. The displacement of the droplet reached 4.7 mm at a laser power of 629 mW. Although this study specifically investigated the phenomenon using a 520 nm green-focused laser beam, the results may be applicable to other scenarios, such as when using light sources of different wavelengths, droplets of various liquid types, or different substrates, for example, droplet motion was also observed on glass and aluminum substrates coated with superhydrophobic coating (Figure S13). Additionally, we observed that the initial part of the first phase of motion can be repeated several times under controlled laser conditions. The droplet can also undergo multiple small displacements when exposed to short laser pulses before the first phase fully develops, as shown in Video S5.

## Materials and Methods

### Experimental Equipment

A green laser (S3 Krypton Series, Wicked lasers, Hong Kong) with a wavelength of 520 nm, focused by a convex lens (49–853, Edmund Optics, USA), was used as a traction beam to induce the motion of the dyed droplets. The dyed droplets were deposited on the superhydrophobic substrate manually by using a syringe. The adjustment of the substrate in the *x*, *y*, and *z* directions was carried out using a custom-built 3-axis precision motorized platform, with precision in the *x* direction being 0.5 μm (M-414.2PD, Physik Instrumente, Germany), and in the *y*-, *z*-directions being 0.25 μm (M-111.1DG, Physik Instrumente, Germany). The dyed droplet motion was recorded using two digital cameras (BFLY-U3-23S6C-C, Edmund Optics, USA), each through a zoom lens (VZM 600i, Edmund Optics, USA), one observing the experiments from the bottom through the transparent substrate and another from the side. The experiment was illuminated using a light source (MI-150, Edmund Optics, USA). A custom-built software program was used to control the motorized platform and record camera videos. A schematic diagram of the experimental setup is shown in Figure S14.

### Substrate Preparation

First, alcohol was sprayed onto a transparent 2 mm thick acrylic plate. The substrate was then gently wiped with cleanroom wipes (Xin Silicon Valley, Aladdin, China). The cleaned substrate was rinsed with deionized water (MFCD00011332, Aladdin, China). An air compressor was used to remove water on the substrate. Finally, a superhydrophobic coating (SOFT99, Glaco, Japan) was sprayed onto the substrate and left to dry in a covered Petri dish for 24 h.

### Droplet Preparation

The droplet was prepared manually using deionized water (MFCD00011332, Aladdin, China) and dyed using blue ink (INK-30, Pilot, Japan) at a volume ratio of 200:1. In preliminary tests, we evaluated 100:1, 200:1, 400:1, 600:1, and pure deionized water. During 5 s of laser irradiation, the 100:1 ratio droplet exhibited greater displacement (Figure S15). However, we selected the 200:1 ratio for further experiments due to the darker solid/liquid interface of the 100:1 ratio droplet, which hindered observation. The droplet was manually deposited onto the superhydrophobic substrate with a syringe.

### Laser Power Testing

A power meter (PM400, Thorlabs, USA) and a thermal power probe (S425C, Thorlabs, USA) were used to measure the power of the laser. For each of the three power levels of the laser, six repetitions of measurements were conducted. The measured laser powers are given in Table S2.

### Determination of the Focal Point Position

#### Determination of the Focal Distance

The distance between the convex lens and the substrate edge was measured using a vernier caliper, based on the lens’s focal length. This position was marked in custom-built software as the focal distance. Before each experiment, the droplet’s leading edge was aligned with the marked point.

#### Determination of the Focal Point on the Substrate

The laser was activated, and the beam height was adjusted until it illuminated the substrate surface. This position was marked in custom-built software as the reference for beam adjustment.

#### Determination of the Initial Droplet Position

After the above steps, a droplet was deposited on the substrate and the laser was activated to ensure the beam passed through the droplet center. The beam’s path was marked in custom-built software to establish the droplet center position. This mark, combined with the focal distance, determines the initial droplet position.

#### Determination of the Focal Position

The substrate height was adjusted along the negative *z*-axis to achieve focal illumination at different positions (top, middle, bottom) on the droplet’s front surface.

### Parameter Analysis

The contact angles of the dyed droplet, including the advancing contact angle θ_a_, the receding contact angle θ_r_, and the equilibrium contact angle θ_e_, were measured using the MB-Ruler tool from camera images. The equilibrium contact angle θ_e_ was calculated as the average of the two contact angles on both sides of a sessile droplet. The measured θ_e_ values are listed in Table S3.

The height *h*, long axis *l*_d_, and the distance between the point where the laser hit the front of the dyed droplet and the substrate *h*_f_ were recorded using a side-view camera. The long axis *l*_l_ and the short axis *l*_s_ of the triple-phase contact line of the droplet were recorded by using a bottom camera. These data were manually measured using MATLAB software.

The propagation path of the refracted beam after the laser hit the front surface of the dyed droplet was predicted using the equation *n*_1_·sin θ_1_ = *n*_2_·sin θ_2_, where *n*_1_ and *n*_2_ represent the refractive indices of air and water, respectively, and θ_1_ and θ_2_ represent the angles of incidence and refraction with respect to the normal. The distance between the refracted beam hitting the rear surface of the droplet, *h*_r_, and the substrate was manually measured with SolidWorks software. Note that the refractive index of water varies by less than 0.016 across different visible light wavelengths^21^ or temperatures,^22,23^ which is ∼1% of the refractive index value 1.33 of water at 20 °C for visible light. Therefore, the influence of dye on the refractive index of water droplets is neglected in the study.

The midpoint of the long axis *l*_d_ of the droplet was treated as the reference point for tracking dyed droplet displacement, which was used to estimate the displacement of droplet *s*. The velocity *u* was calculated by using numerical differentiation of *s*. The line charts of Figures 2, 4, and 5 in this article were smoothed using the smooth function and the Savitzky–Golay (sgolay) method in MATLAB software to remove measurement noise. Based on the displacement curve of the dyed droplet obtained through smoothing, the response time and settling time were calculated using MATLAB software.

## Supporting Information Available

Evolution of the laser path inside the droplet; spatiotemporal evolution of the path of the laser beam during the droplet motion; size evolution of condensed satellite droplets; droplet motion induced by contact angle hysteresis and coalescence; the bubbles burst and pull the droplet toward the light source; displacement distribution of droplets under varying laser power and hit points; the temporal evolution of the droplet from the experiment at different laser powers; effect of laser power on droplet dynamics; the influence of laser power on the droplet; droplet contact angle hysteresis over time at different focal positions; the influence of laser focusing point on the droplet; schematic illustration of the experimental setup; proportion of bubbles bursting from the front side of the droplet; measurement of laser power at different power level; and equilibrium contact angle of droplets (PDF)
Side-view and bottom-view video of droplet motion (MP4)
Part of the bubbles pulling the droplet (MP4)
Influence of laser power on droplet motion (MP4)
Influence of focusing point on droplet motion (MP4)
Repeated droplet motion using short laser pulses (MP4)
