# Supplementary material for: Laser-Induced Photothermal Pulling of Dyed Droplets on a Superhydrophobic Surface
Source: Langmuir. 2025 Apr 29;41(18):11374–81. doi: 10.1021/acs.langmuir.5c00160 (PMC12080327; doi:10.1021/acs.langmuir.5c00160)
Supplement: Supplementary file 1 — la5c00160_si_001.pdf [file la5c00160_si_001.pdf]

Supporting Material

Laser-Induced Photothermal Pulling of Dyed Droplets on Superhydrophobic Surface

Peiying Han,[1] Zhaofei Zhu,[1] Zoran M. Cenev,[2,3*] Ville Liimatainen,[2,4] Heng Zhang,[1] Bo Chang,[1*] Quan Zhou[2*]

[1]School of Mechanical and Electrical Engineering, Shaanxi University of Science and Technology, Xi'An, Shaanxi 710021, China

[2]Department of Electrical Engineering and Automation, School of Electrical Engineering, Aalto University, 02150 Espoo, Finland

[3]Department of Mechanical and Production Engineering, Aarhus University, 8200 Aarhus N, Denmark

[4] ASM Microchemistry Oy, 00560 Helsinki, Finland

[*]Corresponding authors

Supporting Video

Video S1 (Video S1. mp4) shows the side-view and bottom-view video of droplet motion.

Video S2 (Video S2. mp4) shows part of the bubbles pulling the droplet.

Video S3 (Video S3. mp4) shows the influence of laser power on droplet motion.

Video S4 (Video S4. mp4) shows the influence of focusing point on droplet motion.

Video S5 (Video S5. mp4) shows the repeated droplet motion using short laser pulses .

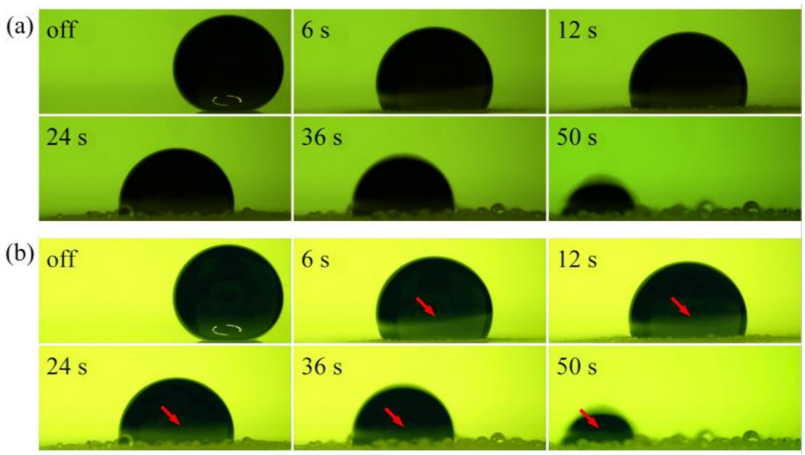

Fig.S1 Evolution of the laser path inside the droplet. (a) Unprocessed side-view images from the experiment. (b) Processed side-view images with enhanced contrast and adjusted histogram to highlight the laser path (indicated by red arrows).

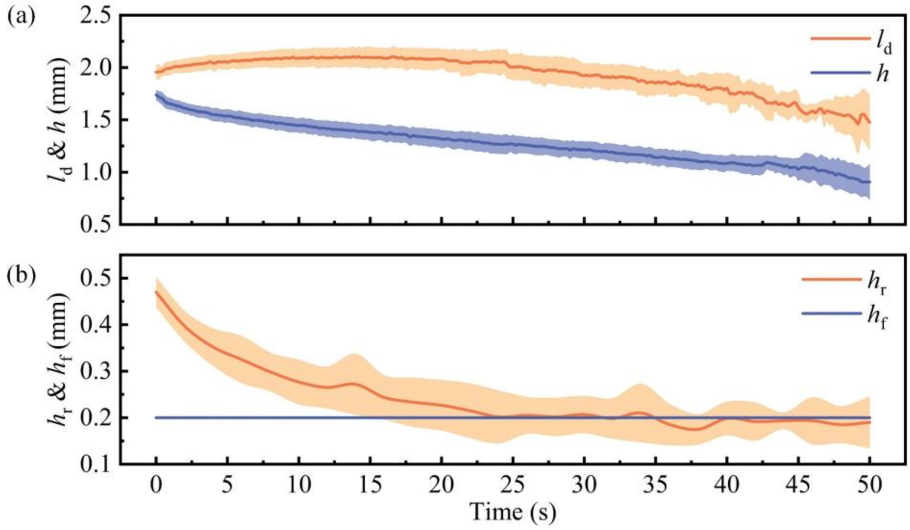

Fig. S2 Statistical analysis of spatiotemporal evolution of the path of the laser beam during the droplet motion (n=5). (a) The evolution of the height h and long axis l_d of the droplet. (b) The evolution of h_r, the distance between the hitting point of the refracted light on the back of the droplet and the substrate within the droplet, and h_f, the distance between the hitting point of the laser beam on the front of the droplet and the substrate. Here, the solid line represents the mean, while the shaded area indicates the standard deviation.

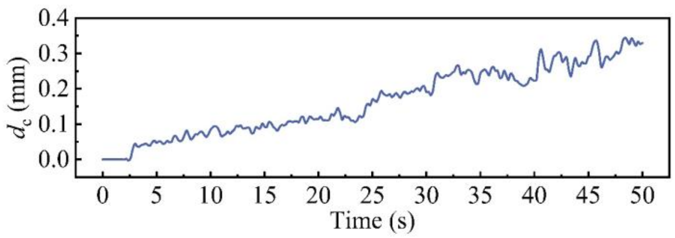

Fig. S3 Size evolution of condensated satellite droplets, with maximum droplet size d_c observed around the triple-phase contact line of the main droplet.

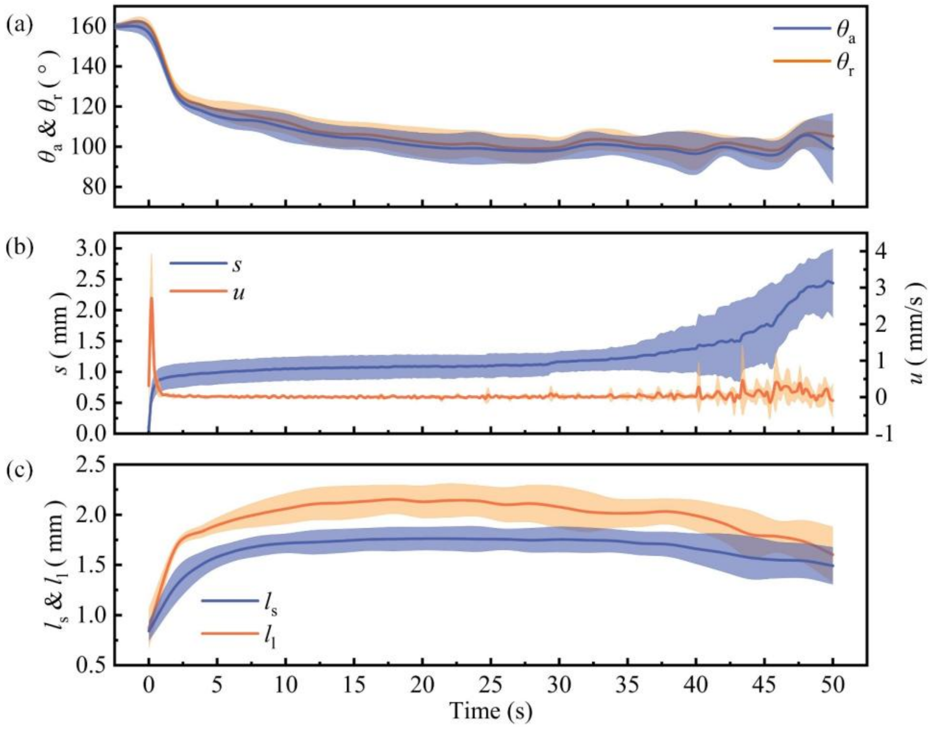

Fig. S4 Statistical analysis of droplet motion induced by contact angle hysteresis and coalescence (n=5). (a) Temporal evolution of the contact angle of the droplet. Here, θ_a and θ_r are the advancing and receding contact angles of the droplet, respectively. (b) The displacement s and velocity u of the droplet through coalescence. (c) Temporal evolution of the shape of triple-phase contact line of the droplet. Here, l_s and l_l are the short and long axes of the triple-phase contact line of the droplet, respectively. Here, the solid line represents the mean, while the shaded area indicates the standard deviation.

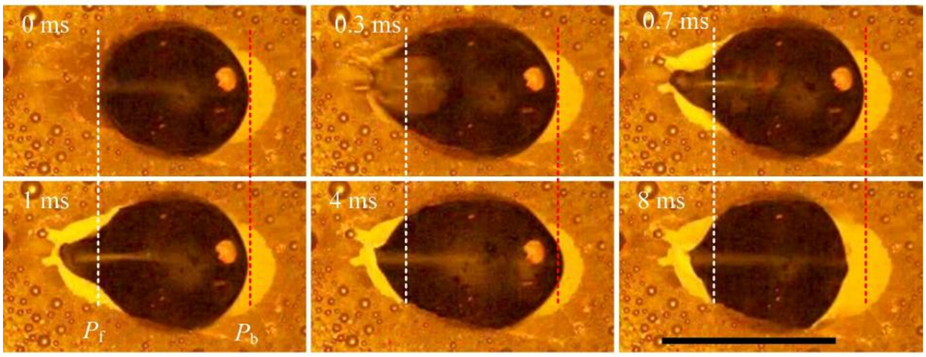

Fig. S5 The bubbles burst and pull the droplet toward the light source. p_f and p_b represent the initial positions of the front and back of the droplet-substrate contact line, respectively. The scale bar is 1 mm. The power of laser is 458 mW. The images were captured by a high-speed camera (Miro-R-311, Phantom, America) with a frame rate of 3000 fps.

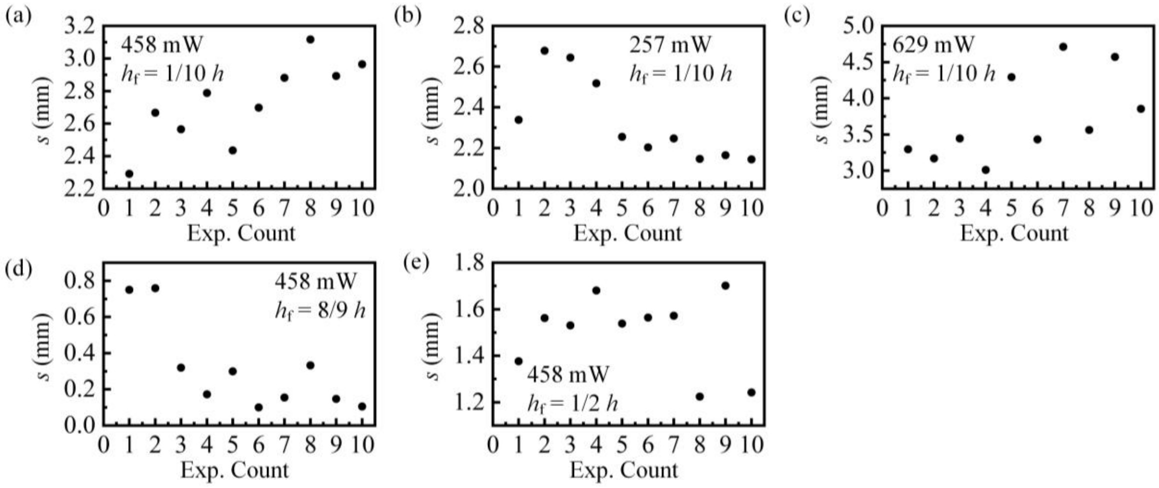

Fig. S6 Displacement distribution of droplets under varying laser power and focus points. (a) Displacement distribution at 458 mW, $h_f = 1/10\ h$. The h_f is the distance between the focus point of the laser beam on the front of the droplet and the substrate, and h is the height of the droplet. (b) Displacement distribution at 257 mW, $h_f = 1/10\ h$. (c) Displacement distribution at 629 mW, $h_f = 1/10\ h$. (d) Displacement distribution at 458 mW, $h_f = 8/9\ h$. (e) Displacement distribution at 458 mW, $h_f = 1/2\ h$. Each experimental set was repeated ten times for statistical relevance.

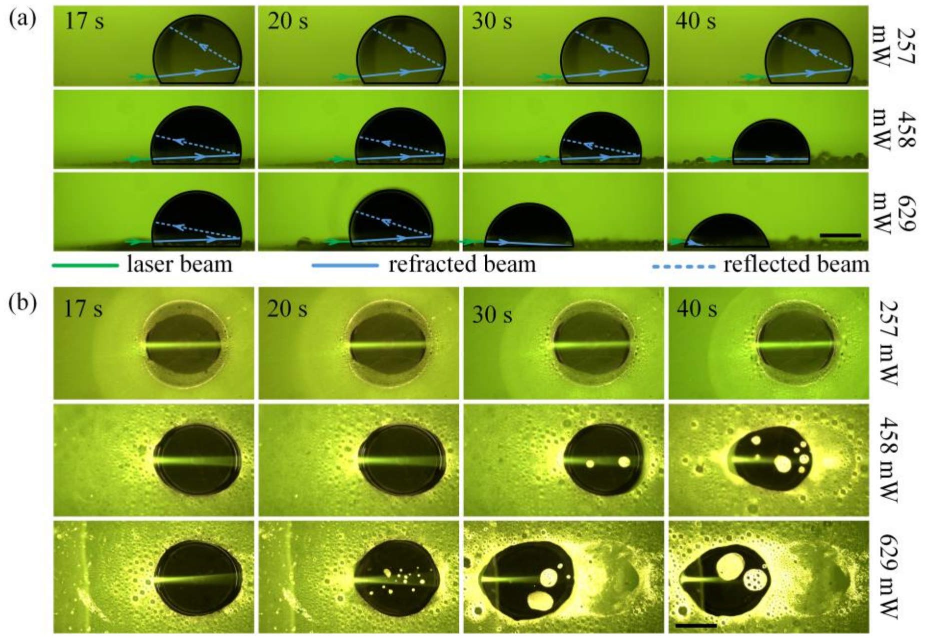

Fig. S7 The temporal evolution of the droplet from the experiment at different laser powers. (a)The inferred path of the laser within the droplet from the bottom-view images experiment. (b) Bottom-view images of the temporal evolution of the solid-liquid interface of the droplet from the experiment. The scale bar is 1mm.

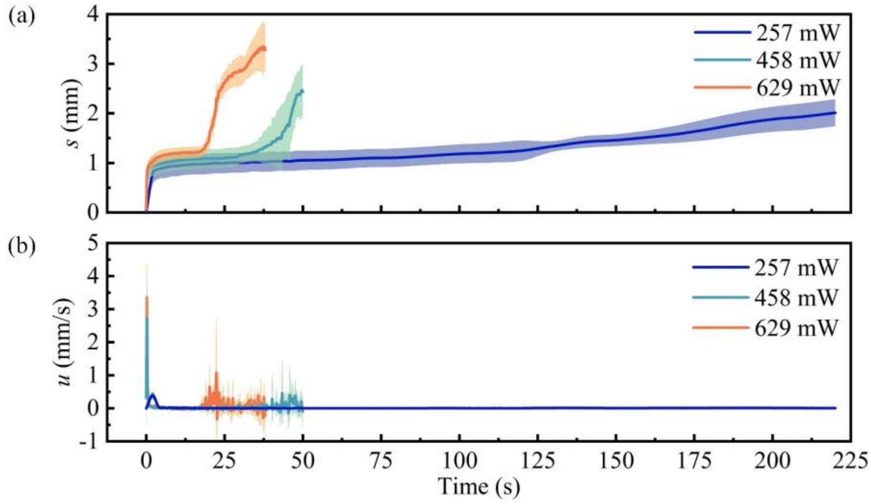

Fig. S8 Statistical analysis of effect of laser power on droplet dynamics (n=5). Temporal evolution of (a) droplet displacement s, and (b) droplet velocity u. The solid line represents the mean, while the shaded area indicates the standard deviation.

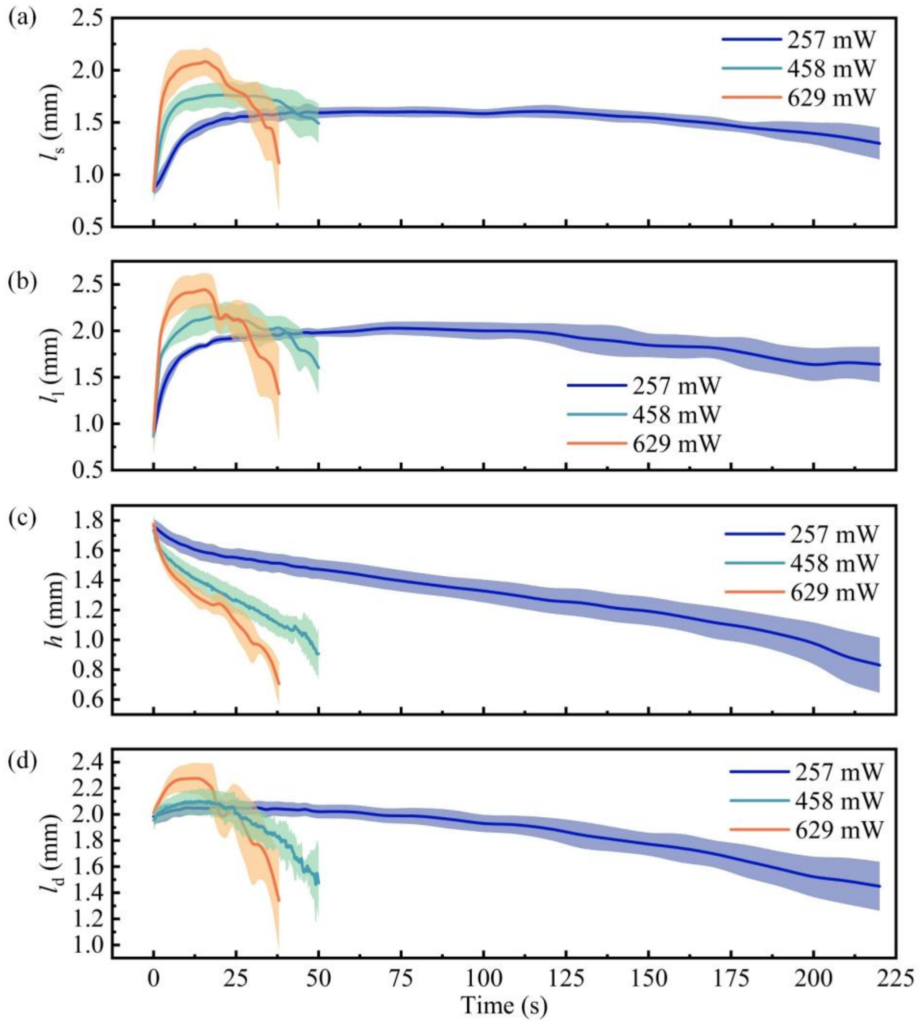

Fig. S9 Statistical analysis of the influence of laser power on the droplet (n=5). (a) Temporal evolution of the short axis of the triple-phase contact line of the droplet l_s. (b) Temporal evolution of the long axis of the triple-phase contact line of the droplet l_l. (c) Temporal evolution of the height of the droplet h. (d) Temporal evolution of the long axis of the droplet l_d. Here, the solid line represents the mean, while the shaded area indicates the standard deviation.

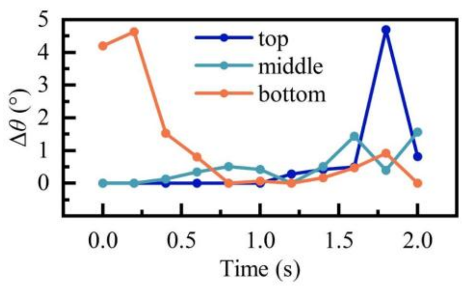

Fig. S10 Droplet contact angle hysteresis over time at different focal positions. $\Delta\theta$ represents contact angle hysteresis.

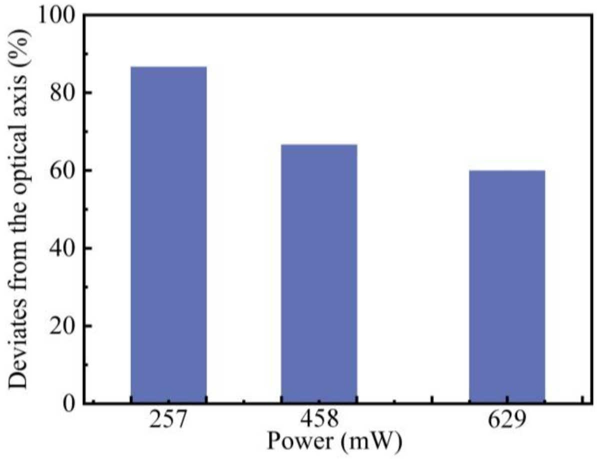

Fig. S11 Probability of droplet deviation from the optical axis. After focused laser irradiation at different power levels near the top surface of the droplet, the probability of the droplet deviating from the optical axis during motion. The experiment was repeated 15 times.

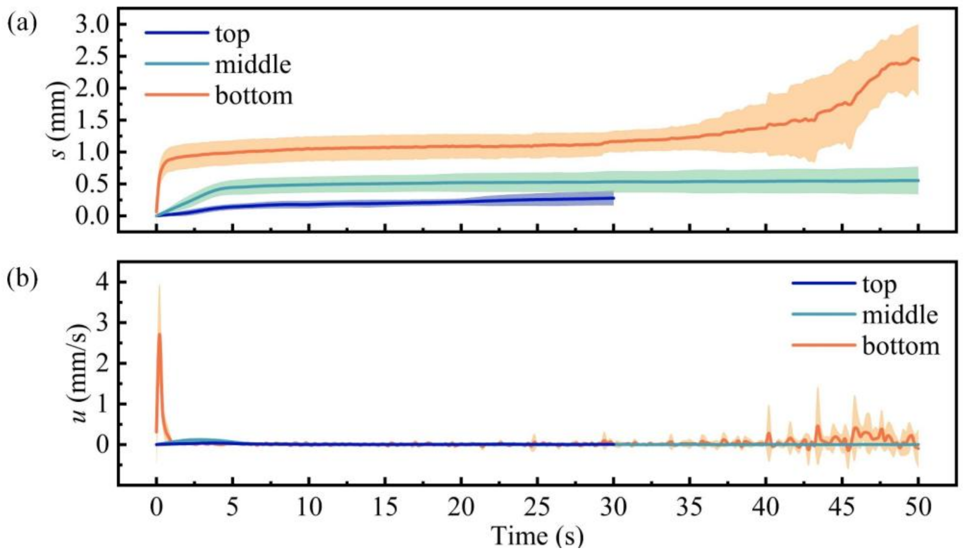

Fig. S12 Statistical analysis of the influence of laser focusing point on the droplet (n=5). (a) The temporal evolution of the displacement of the droplet s. (b) Temporal evolution of the velocity of the droplet u. Here, the solid line represents the mean, while the shaded area indicates the standard deviation.

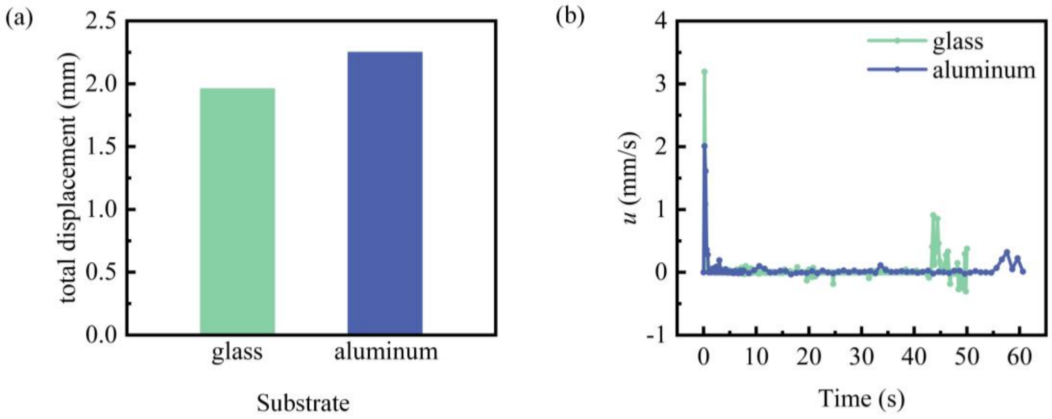

Fig. S13 Droplet motion on various substrates. (a) Total displacement of the droplet. (b) Velocity of the droplet.

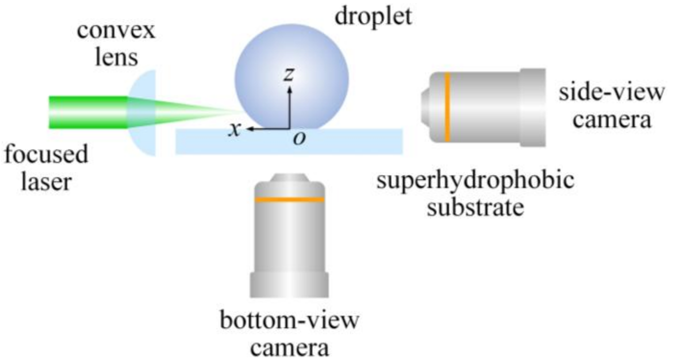

Fig. S14 Schematic illustration of the experimental setup. The positive direction of the x-axis indicating the direction of droplet motion.

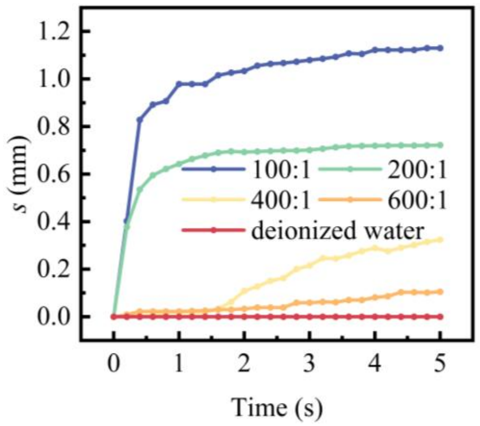

Fig. S15 Impact of different volume ratios of deionized water to ink on droplet displacement

Table S1 Proportion of bubbles bursting from the front side of the droplet

Repetition No.	1	2	3	4	5
Stage1	10 (13)	7 (14)	7 (10)	9 (12)	5 (10)
Stage2	17 (21)	15 (21)	12 (14)	9 (11)	19 (22)

The numbers in parentheses in the table denote the total bubbles that burst from the droplet surface at each stage, while the numbers outside indicate those that burst from the front side of the droplet.

Table S2 Measurement of laser power at different power level (mW)

Power lever \ Repetition No.	1	2	3	4	5	6
257 mW	256	258	258	256	256	256
458 mW	457	458	457	459	459	459
629 mW	630	628	630	629	628	630

Table S3 Equilibrium contact angle of droplets

Repetition No.	1	2	3	4	5
θ_e (°)	160.4	160.1	159.8	159.9	160
